# Determinants of regular self-monitoring of blood pressure in a digital health-based management program among Korean adults living in a remote community : a community-based observational study

**DOI:** 10.1186/s12889-026-26974-5

**Published:** 2026-04-15

**Authors:** Heejung Lee, Juhwan Oh, Jin-Seok Lee

**Affiliations:** 1 Department of Health Policy and Management, Seoul National University College of Medicine, Seoul, Republic of Korea; 2 Department of Medicine, Seoul National University College of Medicine, Seoul, Republic of Korea; 3 Institute of Health Policy and Management, Medical Research Center, Seoul National University, Seoul, Republic of Korea

**Keywords:** Hypertension, Self-monitoring of blood pressure, Digital health, Adherence, Health behaviors, Socioeconomic factors, Community-based intervention

## Abstract

**Background:**

Although recent efforts to integrate digital technologies have accelerated more effective and efficient hypertension management, low adherence and high dropout rates have been reported in real-world digital health programs. This study aimed to identify the factors associated with regular self-monitoring of blood pressure (SMBP) by analyzing real-world app-recorded data from a digital health-based program.

**Methods:**

Data from 186 participants enrolled in a smart online-to-offline digital healthcare program between January and December 2024 were analyzed. Sociodemographic characteristics, health status, and health behaviors of the participants and their regular SMBP performance over 12 weeks and average daily measurement frequency were analyzed. Logistic regression was used to examine the regular SMBP performance; multiple linear regression was performed on the log-transformed average measurement frequency.

**Results:**

Females (odds ratio [OR] = 4.41), unmarried participants (OR = 5.06), and those with a monthly income of 1–2 million KRW (USD 679–1,358) (OR = 3.28) had significantly higher odds of irregular SMBP. Past or current smokers also had higher odds of poor adherence. Participants with self-rated health as “fair” showed a significantly lower measurement frequency than those who rated their health as “good” (− 0.37 times). Those residing farther from the service support center had higher odds of irregular monitoring (OR = 5.12) and fewer measurements per day (− 0.34 times) than those living nearby.

**Conclusion:**

SMBP adherence to digital health programs was lower among socioeconomically and medically vulnerable populations. Physical accessibility remains a significant factor, even in the digital environment. These findings highlighted the need for tailored support strategies to ensure equal participation in digital health interventions.

**Supplementary Information:**

The online version contains supplementary material available at 10.1186/s12889-026-26974-5.

## Background

According to a report by the World Health Organization, approximately 1.3 billion adults aged 30–79 years worldwide have hypertension; however, only 21% have achieved adequate blood pressure (BP) control [1, 2]. Hypertension remains a leading cause of cardiovascular disease, stroke, and kidney disease, and is one of the foremost contributors to premature mortality worldwide [1]. ​ While lifestyle modifications such as diet and exercise are strongly recommended alongside pharmacological treatment [3], self-monitoring of blood pressure (SMBP) is increasingly being recognized as a key strategy in hypertension self-management and control [4].

SMBP, defined as the measurement of blood pressure by individuals outside the clinical setting, typically at home, was highlighted by a joint policy statement from the American Heart Association and American Medical Association as having substantial potential to improve the diagnosis and management of hypertension [5, 6]. Home BP readings enable the detection of white-coat, masked, and resistant hypertension, and provide a more accurate assessment of BP control in patients receiving antihypertensive therapy. This approach supports treatment adherence, encourages patient engagement, and improves BP control rates [7–9]. Moreover, home BP monitoring is considered a better predictor of target organ damage and cardiovascular outcomes than clinical measurements and offers cost-effectiveness benefits in the long term [10]. Notably, regular SMBP has been associated with improved blood pressure control compared with sporadic monitoring [7, 11].

With recent advancements in digital health technologies, SMBP has been integrated into mobile health (mHealth) platforms to facilitate real-time monitoring, data visualization, and interactive interventions [12]. These digital interventions have demonstrated effectiveness in improving BP control [12–14], and meta-analyses have suggested that combining SMBP with digital tools and healthcare provider engagement yields even greater benefits [15]. Digital technologies also promote self-monitoring behavior [16], enhance patient access to personal health data, and help overcome geographic and temporal barriers to chronic disease management.

SMBP in the digital health context still depends heavily on patient engagement and may be hindered by disparities in digital literacy and access. Individuals from socioeconomically disadvantaged groups may face additional challenges when using these digital tools, potentially exacerbating existing health disparities. In Korea, various digital health interventions have been piloted, but studies have reported low engagement and high dropout rates beyond the initial two months, often due to difficulties with device operation and data input [17, 18]. However, most previous studies have relied on self-reported surveys to examine factors associated with SMBP. There is a lack of research using simultaneous measurement of risk factors and SMBP data during study participation period in digital health programs. Therefore, this study aimed to identify the factors associated with regular SMBP practice by analyzing real-world app-recorded data from a digital health-based chronic disease management program. This study was conducted in a rural Korean county with a population of approximately 40,000 (a rapidly aging population), and limited healthcare infrastructure, an environment in which digital health solutions may offer critical support.

## Methods

### Data description

This study utilized data from participants enrolled in the “Development and Demonstration of a Digital Health-Based Online-to-Offline (O2O) Service Model for Chronic Disease Management” conducted over a one-year period between January and December 2024. The O2O service was implemented in Pyeongchang-gun, Gangwon-do, a rural district where access to healthcare is limited, and the population is predominantly older. Participants were provided with Bluetooth-enabled blood pressure monitors or glucometers depending on their condition (or both devices if they had comorbid hypertension and diabetes) and were instructed to use a mobile application for daily self-monitoring, which was encouraged as the optimal monitoring behavior.

The application offers access to educational videos, behavioral intervention messages, and additional offline education sessions, enabling an integrated online and offline chronic disease management experience.

Participants were recruited through public and private healthcare providers in Pyeongchang-gun, including the county’s public health center, three private clinics, several local health posts, and community outreach. Eligible participants were adults aged ≥ 18 years who owned a smartphone and had (1) a prior clinical diagnosis of hypertension or diabetes (including prehypertension or prediabetes), or (2) no previous diagnosis but visited healthcare facilities due to suspected hypertension or diabetes and were subsequently identified as having prehypertension, hypertension, prediabetes, or diabetes based on baseline clinical assessments.

Prehypertension and hypertension were defined based on baseline systolic and diastolic blood pressure thresholds (systolic BP ≥ 130 mmHg or diastolic BP ≥ 80 mmHg), and prediabetes and diabetes were defined using baseline hemoglobin A1c levels (HbA1c ≥ 5.7%).

Sociodemographic, behavioral, and health-related information such as age, sex, education, occupation, lifestyle, and dietary habits were collected through an online questionnaire distributed via text messages. Baseline physician assessments, physical examinations, and clinical test results were obtained. After completing baseline screening, participants installed the “Value Health” app (KachiGeongang) linked to a clinician-facing web portal and were provided with a Bluetooth-enabled BP monitor (OMRON HEM-7142T2). Participants received education on how to use the mobile application, how to measure blood pressure at home, how measurement data were linked to and entered into the application, and how the collected data could be utilized for self-monitoring and follow-up care. Blood pressure measurements obtained during home self-monitoring using the Bluetooth-enabled device were automatically transferred to the Value Health mobile application when the application was activated. Automatic transmission via Bluetooth was the primary mode of data collection in this study, and manual data entry was permitted only in limited circumstances when automatic synchronization was not feasible. Participant registration followed the O2O protocol guidelines established in a previously published protocol study [19]. This study was approved by the Institutional Review Board of Seoul National University Hospital (IRB No. C-2308-183-1463). All participants provided written informed consent prior to participation.

### Study sample

The analytical sample consisted of participants enrolled in the O2O program who met predefined eligibility criteria relevant to the assessment of blood pressure self-monitoring behavior.

The sample for this study included participants with either a clinical or preclinical history of hypertension (or systolic BP ≥ 130 mmHg or diastolic BP ≥ 80 mmHg at baseline) who had completed installation of the “Value Health” application.

To ensure a minimum follow-up period required for defining regular self-monitoring behavior, only data up to October 2024 were included, corresponding to the completion of at least 12 weeks of follow-up for participants enrolled as late as July 30, 2024. Accordingly, the monitoring period varied by participant, ranging from 3 to 9 months, depending on the date of app installation.

Participants who did not meet the minimum follow-up requirement or whose monitoring data could not be evaluated within a consistent observation window were excluded from the present analysis.

Among participants with comorbid hypertension and diabetes, some received blood pressure monitors and glucometers at different time points during program participation. Because self-monitoring behavior may differ between individuals who monitor a single condition and those who monitor multiple conditions, variation in device provision timing could introduce heterogeneity in blood pressure self-monitoring patterns. Therefore, participants with asynchronous device provision were excluded from the present analysis.

These exclusions were applied solely for analytical purposes and do not indicate withdrawal from the program itself.

This study did not perform an a priori sample size calculation, as it represents a secondary observational analysis focusing on determinants of self-monitoring behavior among participants enrolled in a digital health-based chronic disease management program. 

### O2O Services

**Fig. 1:**
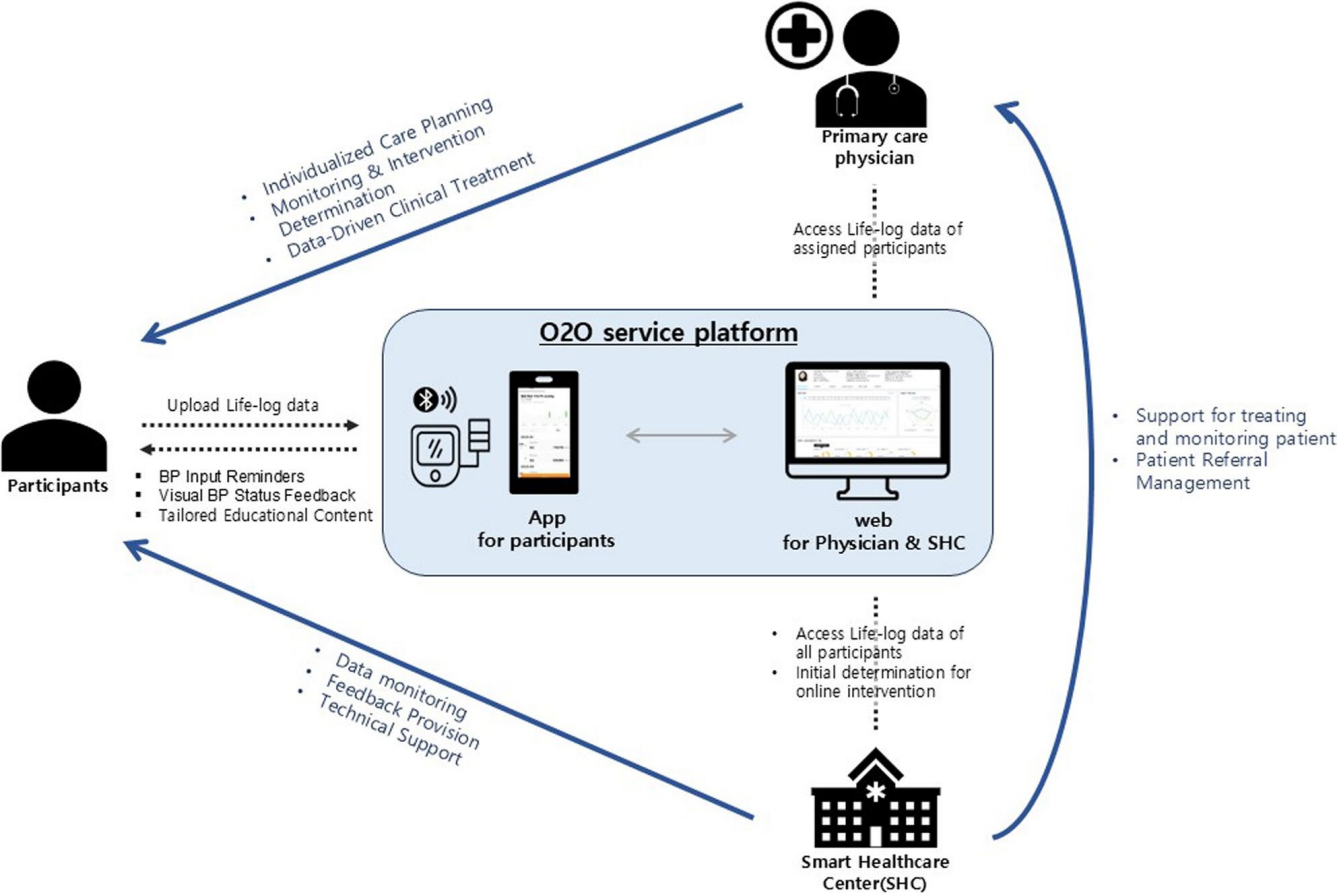
Service and data flow in O2O (Online-to-Offline) service model

The O2O service was structured to support chronic disease management through both online and offline components and was coordinated by the “Smart Health Management Center” affiliated with the Pyeongchang-gun Public Health Center. This center functioned as a community-level organization responsible for the integrated management of individuals with chronic diseases in the region and collaborated with private clinics and community health centersto implement the intervention (Fig. 1).

Participants used the “Value Health” mobile app to enter data on home-monitored BP or blood glucose, as well as lifestyle behaviors such as medication adherence, exercise, and diet. The participants were instructed to measure their BP or blood glucose levels according to individualized targets set by healthcare providers, with at least one measurement per week defined as the minimum adherence threshold of the program. If participants failed to meet the target number of weekly measurements set by their healthcare providers, motivational push notifications were sent via the app.

The application also delivered weekly educational content related to chronic disease management through push messages. A web-based monitoring platform was provided to both staff at the Smart Health Management Center and healthcare providers at affiliated clinics, enabling the Center to oversee participant data at the regional level and allowing healthcare providers to review self-monitoring records of patients registered at their own institutions during routine care or clinic visits. Participants who consistently showed abnormal readings or failed to enter data regularly were contacted via text or phones for additional support and follow-up visits.

### Outcomes and measures

#### Dependent variables

Dependent variables were categorized into two types: (1) regular SMBP and (2) average daily measurement frequency, used to assess participants’ engagement and adherence to SMBP.

Regular monitoring was defined as measuring BP at least once per week for at least 11 of 12 weeks from the date of app installation. In evaluations of hypertension interventions, including digital health–based programs, an observation period of approximately three months is frequently used as a minimum unit to assess intervention effects [12–15],. Based on prior research, a 12-week period was adopted in the present study as a practical minimum duration for defining regular SMBP practice. Participants were then dichotomized into the “irregular monitoring [1]” or “regular monitoring [0]” groups.

The average daily measurement frequency was calculated as the number of cumulative SMBP records divided by the number of participation days using up to 24 weeks (168 days) of data. Unlike regular SMBP practice, which reflects whether participants met a minimum monitoring criterion, average daily measurement frequency represents the intensity and stability of monitoring behavior. Because daily frequency is sensitive to short-term fluctuations, a longer observation window of up to 24 weeks was used to obtain a more stable estimate of monitoring intensity. For participants who engaged for < 24 weeks, the frequency was calculated as follows: (number of measurements)/(number of participation days). Only the first 24 weeks were used for those with more than 24 weeks of data (i.e., measurements over 168 days).

In this study, the SMBP was defined as valid only when both measurements were taken using the device, and the value was successfully transmitted and recorded in the mobile application.

#### Independent variables

The independent variables included sociodemographic characteristics such as sex, age, marital status, education level, residential area, and monthly household income. Income was categorized into four groups based on income distribution: low (< KRW 1 million, < USD 679), lower-middle (KRW 1–2 million, USD 679–1,358), upper-middle (KRW 2–3 million, USD 1,358–2,038), and high (≥ KRW 3 million, > USD 2,038) ; health status such as chronic disease history, family history, medication status, BP control, and body mass index (BMI) and Perceived health status. BMI status was categorized as normal weight (18.5–24.9 kg/m²) and overweight (≥ 25.0 kg/m²). Perceived health status was assessed using a self-reported five-point scale (very good, good, moderate, poor, very poor) and recategorized into three levels: good (very good or good), fair (moderate), and poor (poor or very poor).

Health behavior variables included smoking, alcohol consumption, exercise, and previous self-monitoring. Alcohol consumption was assessed based on weekly drinking frequency and average amount per occasion and classified into low-risk or high-risk drinking groups according to World Health Organization criteria. High-risk drinking was defined as an average of ≥ 7 drinks per occasion for men or ≥ 5 drinks per occasion for women at least once per week, or drinking on two or more occasions per week for women. Exercise status was categorized as regular exercise (at least three times per week), irregular exercise, or no exercise based on self-reported responses. Previous self-monitoring experience was defined as any reported home measurement (≥ 1 time per week), while participants who reported no measurement were classified as having no prior self-monitoring experience.

Among these, residential area was used as an indicator of physical accessibility to the Smart Health Management Center. Specifically, the area was classified based on accessibility to a center located in the Southern part of the region (the Smart Health Management Center at the Pyeongchang Public Health Center). Areas reachable within approximately 20 min by car were categorized as “close,” while Northern regions requiring at least 30 min of driving time were classified as “remote,” indicating reduced accessibility.

### Statistical analyses

Logistic regression analysis was conducted to identify the factors associated with irregular SMBP practice (poor compliance). A multiple linear regression analysis was performed to examine the factors affecting the average daily measurement frequency.

As the distribution of the average daily measurement frequency was right-skewed, a log transformation was applied. For interpretability, the estimates were retransformed to the original scale using Duan’s smearing estimator. All analyses were performed using R version 4.4.1.

## Results

A total of 322 participants were enrolled in the O2O program and completed baseline assessments between January and June 2024. Among these, participants who were enrolled exclusively for diabetes management without blood pressure monitoring (*n* = 22) were excluded from the present analysis, as the study focused on determinants of blood pressure self-monitoring behavior.

**Fig. 2:**
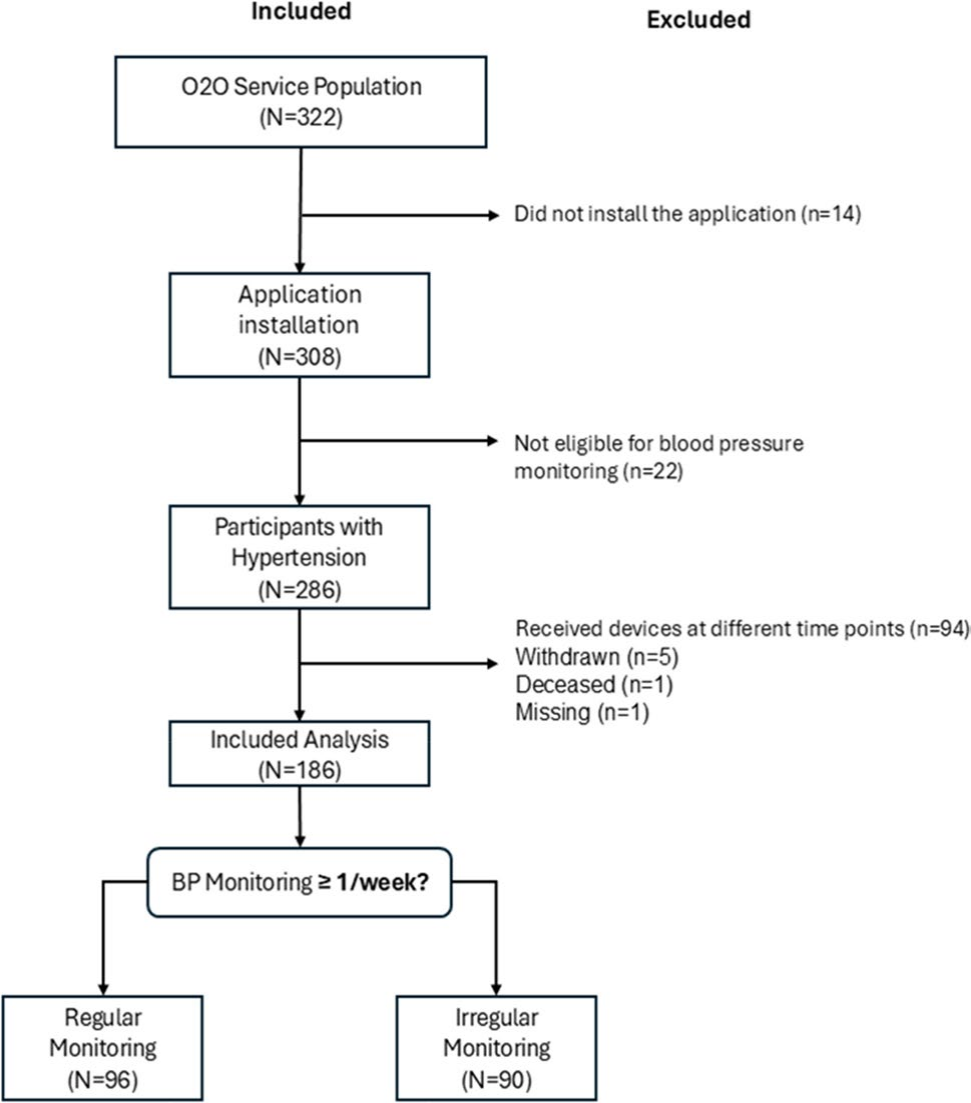
Study population identification process

In addition, participants whose blood pressure monitoring data could not be evaluated within a consistent observation window—primarily due to delayed or non-overlapping initiation of blood pressure monitoring—were excluded for analytical reasons (*n* = 94). After applying these predefined inclusion criteria, the final analytical sample consisted of 186 participants (Fig. 2).

A total of 186 participants were included in the analysis of factors associated with regular SMBP. Sociodemographic characteristics, disease-related conditions, health behaviors, and clinical health statuses were compared between the regular and irregular monitoring groups using univariate analyses (Table 1). Statistically significant differences were observed in monthly income (*p* = 0.030) and geographic access to health centers (*p* < 0.001), whereas no significant differences were found for other examined characteristics.

**Table 1:** Comparison of participant characteristics by regular self-monitoring of blood pressure practice

Characteristic	Regular monitoring (*N* = 96)	Irregular monitoring (*N* = 90)	*p*
Sociodemographic factors			
Age group			0.596
-59	37 (38.5%)	40 (44.4%)	
60–69	41 (42.7%)	32 (35.6%)	
70-	18 (18.8%)	18 (20.0%)	
Sex			0.230
Male	46 (47.9%)	52 (57.8%)	
Female	50 (52.1%)	38 (42.2%)	
Marital status			0.173
Married or cohabitating	80 (83.3%)	82 (91.1%)	
Other	16 (16.7%)	8 (8.9%)	
Education			0.108
Middle school or lower	42 (43.8%)	27 (30.0%)	
High school	29 (30.2%)	29 (32.2%)	
College or higher	25 (26.0%)	34 (37.8%)	
Employment			0.336
Yes	81 (84.4%)	70 (77.8%)	
No	15 (15.6%)	20 (22.2%)	
Monthly income*			**0.030**
Low (< 1 million KRW, < USD 679)	22 (22.9%)	25 (27.8%)	
Middle-low (1–2 million KRW, USD 679–1,358)	29 (30.2%)	11 (12.2%)	
Middle-high (2–3 million KRW, USD 1,358–2,038)	12 (12.5%)	15 (16.7%)	
High (> 3 million KRW, > USD 2,038)	33 (34.4%)	39 (43.3%)	
Access to the center			**0.000**
Close	48 (50.0%)	69 (76.7%)	
Remote	48 (50.0%)	21 (23.3%)	
Health status			
Family history			0.568
None	32 (33.3%)	24 (26.7%)	
Yes	54 (56.2%)	54 (60.0%)	
Etc	10 (10.4%)	12 (13.3%)	
Disease			0.442
hypertension only	26 (27.1%)	30 (33.3%)	
Hypertension & Diabetes	70 (72.9%)	60 (66.7%)	
Medication			0.148
None	24 (25.0)	24 (26.7)	
Hypertension only	42 (43.8%)	48 (53.3%)	
Diabetes only	9 (9.4%)	2 (2.2%)	
Hypertension & Diabetes	21 (21.9%)	16 (17.8)	
BP control^†^			0.772
Well-controlled	14 (14.6)	15 (16.7)	
Moderately controlled	30 (31.2)	24 (26.7)	
Uncontrolled	52 (54.2)	51 (56.7)	
BMI status			0.088
Normal weight	30 (31.2)	40 (44.4)	
Overweight	66 (68.8)	50 (55.6)	
Perceived health			0.600
Good	12 (12.5)	16 (17.8)	
Fair	54 (56.2)	47 (52.2)	
Poor	30 (31.2)	27 (30.0)	
Health behaviors			
Smoking status			0.369
Never	55 (57.3)	57 (63.3)	
Past smoker	26 (27.1)	25 (27.8)	
Current smoker	15 (15.6)	8 (8.9)	
Alcohol consumption			0.645
Low risk	67 (69.8)	59 (65.6)	
High risk	29 (30.2)	31 (34.4)	
Exercise			0.194
Regular exercise	33 (34.4)	39 (43.3)	
Irregular exercise	37 (38.5)	36 (40.0)	
No exercise	26 (27.1)	15 (16.7)	
Previous Self-monitoring			0.567
Yes	44 (45.8)	46 (51.1)	
No	52 (54.2)	44 (48.9)	
Measurement^‡^			0.541
hypertension only	27 (28.1)	30 (33.3)	
Hypertension & Diabetes	69 (71.9)	60 (66.7)	

*BP* Blood pressure, *SBP* Systolic BP, *DBP *Diastolic BP,BMI Body mass index, *KRW *South Korean Won

^†^BP control: Well-controlled (SBP < 130 mmHg and DBP < 80 mmHg), Moderately controlled (130 ≤ SBP < 140 mmHg or 80 ≤ DBP < 90 mmHg), Uncontrolled (SBP ≥ 140 mmHg or DBP ≥ 90 mmHg) ^‡^Measurement: “Hypertension only” refers to participants who receive and use only a blood pressure monitor. “Hypertension & Diabetes” refers to those who receive and use both blood pressure and glucose monitors

* 1 USD = 1,472.5 KRW (2024 government official rate, MOEF)

Boldface indicates statistical significance at *p* < 0.05

Factors associated with irregular SMBP(poor compliance) were identified using a logistic regression analysis conducted using weekly monitoring (< 1 time/week) as the dependent variable (Table 2). After adjustment for all covariates included in the model, among the sociodemographic factors, sex, marital status, monthly income, and geographic access to health centers were statistically significant. Female participants were more likely to exhibit irregular monitoring compared with males (odds ratio [OR] = 4.41, 95% confidence interval [CI] = 1.51–14.19, *p* = 0.009). Participants without a spouse had higher odds of irregular monitoring than those who were married or cohabitating (OR = 5.06, 95% CI = 1.53–18.74, *p* = 0.011). Compared with the lowest income group, those with a monthly income of 1–2 million KRW(USD 679–1,358) had higher odds of irregular monitoring (OR = 3.28, 95% CI = 1.10–10.45, *p* = 0.037). Participants living in remote areas had higher odds of irregular monitoring than those living in close proximity to the center (OR = 5.12, 95% CI = 2.32–12.04, *p* < 0.001). Among health behavior factors, both past smokers (OR = 4.02, 95% CI = 1.28–13.76, *p* = 0.021) and current smokers (OR = 6.02, 95% CI = 1.50–26.81, *p* = 0.014) had higher odds of irregular monitoring compared with never smokers. Participants who were overweight showed a tendency toward irregular monitoring compared with those with normal weight, although this was marginally significant (OR = 2.04, 95% CI = 0.92–4.60, *p* = 0.081). The Hosmer–Lemeshow test indicated a good model fit (*p* = 0.131), and the Nagelkerke R² value was 0.352.

**Table 2:** Logistic regression analysis results for irregular self-monitoring blood pressure practice

Predictors	Odds ratio	CI	*p*
Sociodemographic factors			
Age group			
-59	1		
60–69	1.75	0.66–4.69	0.260
70-	0.65	0.19–2.22	0.495
Sex			
Male	1		
Female	4.41	1.51–14.19	**0.009**
Marital status			
Married or cohabitating	1		
Other	5.06	1.53–18.74	**0.011**
Education			
Middle school or lower	1		
High school	0.60	0.23–1.55	0.294
College or higher	0.54	0.17–1.64	0.277
Employment			
Yes	1		
No	0.44	0.16–1.16	0.102
Monthly income*			
Low (< 1 million KRW, < USD 679)	1		
Middle-low (1–2 million KRW, USD 679–1,358)	0.30	0.1–0.91	**0.037**
Middle-high (2–3 million KRW, USD 1,358–2,038)	0.62	0.18–2.06	0.434
High (> 3 million KRW, > USD 2,038)	0.68	0.2–2.24	0.521
Access to the center			
Close	1		
Remote	0.20	0.08–0.43	**0.000**
Health status			
Family history			
None	1		
Yes	0.97	0.42–2.23	0.941
Etc	0.68	0.19–2.36	0.546
Disease			
Hypertensions only	1		
Hypertension & Diabetes	0.80	0.33–1.91	0.617
Medication			
None	1		
Hypertension only	1.00	0.4–2.5	0.994
Diabetes only	4.01	0.65–35.79	0.160
Hypertension & Diabetes	1.69	0.51–5.75	0.397
BP control^†^			
Well-controlled	1	0.27–2.42	0.716
Moderately controlled	1.01	0.32–3.13	0.991
Uncontrolled	0.82	0.27–2.42	0.716
BMI status			
Normal weight	1		
Overweight	2.04	0.92–4.6	0.081
Perceived health			
Good	1		
Fair	2.08	0.7–6.53	0.195
Poor	1.69	0.5–6.01	0.405
Health behaviors			
Smoking status			
Never	1		
Past smoker	4.02	1.28–13.76	**0.021**
Current smoker	6.02	1.5–26.81	**0.014**
Alcohol consumption			
Low risk	1		
High risk	0.83	0.35–1.96	0.676
Exercise			
Regular exercise	1		
Irregular exercise	1.36	0.58–3.25	0.479
No exercise	1.48	0.52–4.29	0.460
Previous Self-monitoring			
Yes	1		
No	1.88	0.84–4.33	0.131

*BP* Blood pressure, *BMI* Body mass index, *KRW* South Korean Won, *CI* Confidence interval

Boldface indicates statistical significance at *p* < 0.05

†BP control: Well-controlled (SBP <130 mmHg and DBP <80 mmHg), Moderately controlled (130≤SBP<140 mmHg or 80≤DBP<90 mmHg), Uncontrolled (SBP ≥140 mmHg or DBP ≥90 mmHg) ‡Measurement: "Hypertension only" refers to participants who receive and use only a blood pressure monitor. "Hypertension & Diabetes" refers to those who receive and use both blood pressure and glucose monitors

* 1 USD = 1,472.5 KRW (2024 government official rate, MOEF)

Notably, while monthly income and geographic access to the center were the only variables showing significant differences in the univariate comparisons (Table 1), the multivariate logistic regression analysis identified additional sociodemographic and behavioral factors—including sex, marital status, and smoking status—as independently associated with irregular SMBP (poor compliance) after adjustment (Table 2).

A multiple linear regression analysis was conducted to examine factors associated with the daily frequency of BP monitoring. Because the outcome variable exhibited a right-skewed distribution, the analysis was performed on a log-transformed scale; for intuitive interpretation, the results were interpreted based on the estimates retransformed to the original scale using smearing retransformation (Table 3). Unlike the logistic regression analysis focusing on minimum adherence (weekly monitoring), this analysis assessed the intensity of engagement reflected by daily monitoring frequency. Geographic access to the center and perceived health status were significantly associated with the daily frequency of BP monitoring (Table 3). Participants living in remote areas exhibited a 0.34 times lower daily monitoring frequency than those living in nearby areas (β=-0.57, *p* = 0.0134). Additionally, individuals who perceived their health as “fair” monitored their BP 0.37 times less frequently compared with those who perceived their health as “good” (β=-0.70, *p* = 0.0351).

**Table 3:** Multiple regression analysis and retransformed results for daily average self-monitoring of blood pressure frequency

Variable	Log-transformed		Smearing retransformation	
	β (SE)	Pr(>|t|)	Frequency (95% CI)^a^	difference^b^
Sociodemographic Factor				
Age group				
-59	-	-	0.7 (0.25–2)	ref
60–69	-0.09 (0.3)	0.771	0.68 (0.24–1.95)	-0.02
70-	0.1 (0.38)	0.7832	0.55 (0.18–1.66)	-0.15
Sex				
Male	-	-	0.73 (0.26–2.12)	ref
Female	-0.52 (0.3)	0.0901	0.58 (0.21–1.68)	-0.15
Marital status				
Married or cohabitating	-	-	0.7 (0.25–2.01)	ref
Other	-0.61 (0.34)	0.0746	0.4 (0.13–1.26)	-0.3
Education				
Middle school or lower	-	-	0.46 (0.16–1.36)	ref
High school	0.33 (0.29)	0.2566	0.7 (0.24–2.07)	0.23
College or higher	0.42 (0.33)	0.205	0.86 (0.31–2.42)	0.4
Employment				
Yes	-	-	0.63 (0.22–1.8)	ref
No	0.25 (0.28)	0.3851	0.79 (0.26–2.4)	0.16
Monthly income*				
Low (< 1 million KRW, < USD 679)	-	-	0.61 (0.22–1.75)	ref
Middle-low (1–2 million KRW, USD 679–1,358)	-0.08 (0.32)	0.8109	0.45 (0.15–1.33)	-0.16
Middle-high (2–3 million KRW, USD 1,358–2,038)	0.12 (0.37)	0.7418	0.82 (0.27–2.52)	0.21
High (> 3 million KRW, > USD 2,038)	0.05 (0.35)	0.8875	0.76 (0.27–2.12)	0.14
Access to the center				
Close	-	-	0.79 (0.28–2.27)	ref
Remote	-0.57 (0.23)	**0.0134**	0.45 (0.15–1.31)	**-0.34**
Health status				
Family history				
None	-	-	0.49 (0.17–1.45)	ref
Yes	0.37 (0.25)	0.1475	0.75 (0.27–2.11)	-0.07
Etc	0.05 (0.36)	0.8895	0.67 (0.21–2.14)	-0.25
Disease				
Hypertension only	-	-	0.89 (0.32–2.54)	ref
Hypertension & Diabetes	-0.38 (0.26)	0.1466	0.56 (0.2–1.65)	-0.33
Medication				
None	-	-	0.76 (0.27–2.2)	ref
Hypertension only	-0.21 (0.27)	0.4366	0.7 (0.25–1.99)	-0.22
Diabetes only	0 (0.51)	0.9999	0.48 (0.14–1.64)	-0.21
Hypertension & Diabetes	-0.31 (0.36)	0.3942	0.49 (0.17–1.45)	0.06
BP control^†^				
Well-controlled	-	-	0.73 (0.24–2.25)	ref
Moderately controlled	-0.37 (0.33)	0.2669	0.49 (0.17–1.44)	-0.24
Uncontrolled	-0.01 (0.32)	0.9774	0.73 (0.26–2.07)	0
BMI status				
Normal weight	-	-	0.71 (0.24–2.1)	ref
Overweight	0.08 (0.23)	0.7343	0.63 (0.23–1.8)	-0.08
Perceived health				
Good	-	-	0.95 (0.3–2.98)	ref
Fair	-0.7 (0.33)	**0.0351**	0.58 (0.21–1.61)	**-0.37**
Poor	-0.53 (0.37)	0.1550	0.67 (0.24–1.94)	-0.28
Health Behaviors				
Smoking status				
Never	-	-	0.69 (0.24–1.97)	ref
Past smoker	-0.25 (0.32)	0.44	0.71 (0.25–2.08)	0.03
Current smoker	-0.52 (0.4)	0.1926	0.43 (0.15–1.27)	-0.26
Alcohol consumption				
Low risk	-	-	0.68 (0.24–1.96)	ref
High risk	-0.36 (0.26)	0.1681	0.62 (0.22–1.81)	-0.06
Exercise				
Regular exercise	-	-	0.73 (0.25–2.15)	ref
Irregular exercise	-0.04 (0.26)	0.8671	0.7 (0.25–1.95)	-0.03
No exercise	-0.33 (0.31)	0.2956	0.48 (0.16–1.44)	-0.25
Previous self-monitoring				
Yes	-	-	0.7 (0.24–2.05)	ref
No	-0.31 (0.24)	0.1963	0.63 (0.23–1.79)	-0.06

Boldface indicates statistical significance at *p < 0.05*

†BP control: Well-controlled (SBP <130 mmHg and DBP <80 mmHg), Moderately controlled (130≤SBP<140 mmHg or 80≤DBP<90 mmHg), Uncontrolled (SBP ≥140 mmHg or DBP ≥90 mmHg) ‡Measurement: "Hypertension only" refers to participants who receive and use only a blood pressure monitor. "Hypertension & Diabetes" refers to those who receive and use both blood pressure and glucose monitors

* 1 USD = 1,472.5 KRW (2024 government official rate, MOEF)

*SE* standard error, *CI* confidence interval, *BP* blood pressure, *SBP* systolic BP, DBP, diastolic, *BP* BMI, body mass index, *KRW* South Korean Won

**Fig. 3:**
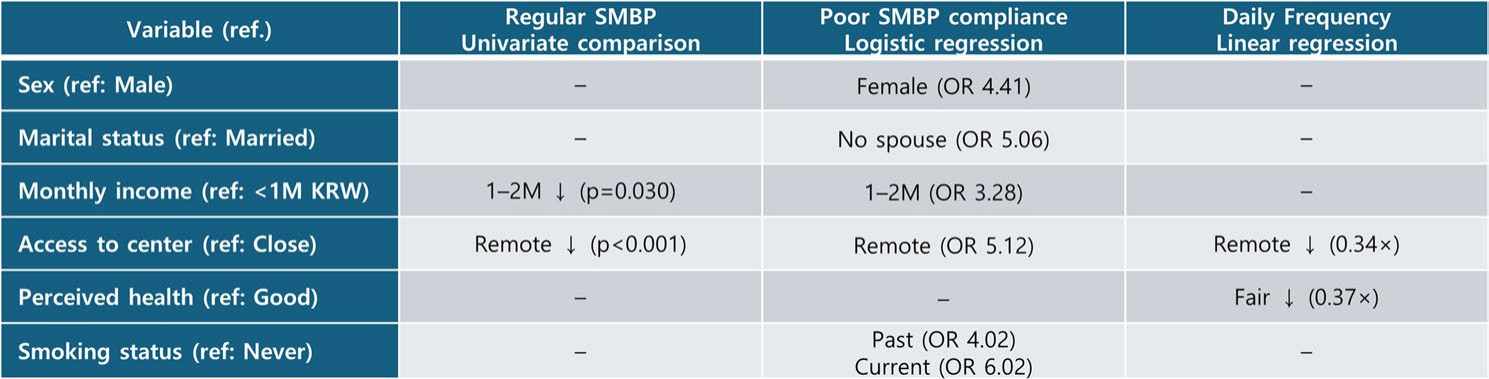
Summary of significant factors associated with regular SMBP practice and daily monitoring frequency across analytical models. Notes. *↓ *indicates a lower likelihood or frequency compared with the reference category. Odds ratios (ORs) are shown for logistic regression analyses, and multiplicative effects (×) represent retransformed estimates from linear regression models. Only variables showing statistical significance (*p* < 0.05) in at least one analysis are presented

Figure 3 summarizes the significant factors associated with regular SMBP practice and daily BP monitoring frequency identified across the univariate, logistic regression, and linear regression analyses.

## Discussion

This study analyzed two related but conceptually distinct outcome variables. Specifically, regular SMBP practice, defined as adherence to a minimum monitoring guideline of at least one measurement per week, and average daily SMBP frequency, which reflects the activeness or intensity of self-monitoring behavior, were examined separately as outcome measures.

The findings of this study demonstrated that sociodemographic factors such as sex, marital status, income level, and geographic accessibility, as well as perceived health and certain health behaviors, were significantly associated with regular performance and frequency of SMBP. Female participants, those without a spouse, and those in the lower-middle income group (KRW 1–2 million, USD 679–1,358) were more likely to exhibit irregular SMBP (poor compliance). In terms of health behaviors, both former and current smokers were more likely to exhibit irregular SMBP compared with non-smokers, with the lowest adherence observed among current smokers. These results indicate that individuals from vulnerable populations who may require more proactive chronic disease management tend to be less engaged in self-monitoring practices. In particular, both socioeconomic disadvantages and unhealthy behaviors were associated with a higher likelihood of irregular SMBP.

Previous studies on self-management suggested that women, older adults, and individuals with higher educational attainment tend to exhibit better health behaviors [20]. However, this study found that women were significantly more likely to exhibit irregular SMBP. This result is consistent with those of previous studies reporting lower SMBP adherence among women, those with lower incomes, and those with lower educational attainment in US [7]. This pattern may reflect broader differences in health behavior and self-management engagement shaped by socially constructed gender roles. Men have been reported to favor autonomy-preserving or self-directed health strategies in certain contexts, whereas women—particularly in rural or socioeconomically vulnerable populations—may experience greater caregiving burdens and competing role demands that constrain sustained engagement [21, 22]. These findings may also reflect the characteristics of digital-based SMBP, as a recent Korean study found that younger male users are more likely to utilize mHealth tools [23]. In addition, persistent gender disparities in digital access and literacy may further influence engagement with digital health tools [24]. Although these mechanisms were not directly assessed in this study, they may partly explain the observed differences in SMBP adherence.

Notably, the factors associated with the two outcome variables were not identical. Sociodemographic characteristics such as sex and marital status were significant predictors of irregular SMBP practice but showed only marginal or non-significant associations with average daily monitoring frequency. This finding suggests that social and household contexts may influence whether individuals initiate and maintain SMBP at a minimum recommended level, whereas the intensity or frequency of monitoring may be more strongly influenced by individual perceptions and environmental constraints.

In contrast, perceived health status was not significantly associated with irregular SMBP practice but was significantly related to average daily monitoring frequency. Participants who perceived their health as “fair” tended to monitor their blood pressure less frequently than those who perceived their health as “good,” indicating that subjective health perception may influence motivation or engagement intensity rather than the mere performance of routine monitoring.

Importantly, geographic access to the Smart Health Management Center emerged as a consistently significant factor across both outcome measures, suggesting that accessibility functions as a structural determinant affecting both the likelihood of regular SMBP practice and the frequency of self-monitoring.

Participants living in remote areas farther from the Smart Health Management Center were significantly more likely to exhibit irregular SMBP and had a lower average monitoring frequency. Although one of the primary goals of digital healthcare is to overcome geographic barriers, these findings suggest that physical accessibility and support remain critical components of service utilization even in rural population. Considering that the study population primarily resided in rural areas with a high proportion of older adults, such geographic disparities were particularly pronounced. SMBP not only required device use but also data entry through a smartphone application, which likely imposed additional digital literacy demands. Participants with poor monitoring adherence often reported difficulties such as device disconnection or application login errors.

To address these barriers, the Smart Health Management Center, located within the Pyeongchang County Public Health Center, provided face-to-face education and technical support. However, for residents living in Northern remote regions, it took over 60 min by car to reach the center, and in-person follow-up by the research staff was limited. These logistical differences may have contributed to disparities in engagement, suggesting the need for complementary physical support structures in the digital healthcare service design.

Taken together, these findings underscore that regular SMBP practice and monitoring frequency capture different dimensions of self-management behavior and should be interpreted as complementary rather than interchangeable indicators.

Additionally, among participants who remained in the program for > 6 months, an analysis of the continuity of measurement patterns revealed that 77.4% maintained their SMBP pattern from weeks 1 to 12 through weeks 13–24 (Cohen’s κ = 0.559) (Supplement 1). This suggests that the self-monitoring habits established during the early phase of the intervention were likely sustained over time, underscoring the importance of providing intensive education and support at the beginning of the program. However, because this study did not conduct a long-term follow-up, further research is needed to validate the persistence of SMBP behavior over extended periods.Social determinants of health and social determinants digital health often overlap substantially [25]. The Organisation for Economic Co-operation and Development has also highlighted user-level barriers to digital health adoption, including lack of education, digital illiteracy, and delayed feedback mechanisms [26]. Paradoxically, those who stand to benefit the most from digital technologies may face the greatest challenges in accessing and utilizing them. The vulnerability characteristics associated with irregular SMBP identified in this study may have a stronger impact on individuals with limited digital access. This suggests that populations already vulnerable in terms of health may face dual disadvantages owing to the digital divide, thereby amplifying health inequities in the context of digital health.

This study empirically highlighted the intersection of health disparities and the digital divide in self-monitoring practices within the digital healthcare framework. Effective management of hypertension and prevention of its complications require a coordinated approach involving health systems, healthcare providers, and patients [11]. Furthermore, for the successful dissemination and sustainability of hypertension management using digital healthcare, it is essential to ensure that users trust digital technologies and are supported in learning and utilizing them effectively. Governments should adopt an equity-oriented approach from the early stages of digital health policy [27]. Continuous policy support is needed to enhance individuals’ digital literacy in line with the advancement of digital innovation. Through such measures, digital healthcare can be systematically prepared to deliver equitable benefits to all population groups, rather than exacerbating existing health disparities.

This study is significant in that it empirically identified factors influencing irregular SMBP using real-world application records of participants enrolled in a digital health-based chronic disease management program. A notable strength of this study is its use of actual behavioral data, rather than relying solely on self-reported surveys, thereby providing a more reliable evaluation of SMBP.

However, this study has several limitations. First, most independent variables were collected via online questionnaires, which may have been influenced by participants’ mobile device literacy or Internet access, potentially affecting the reliability of the responses. Second, digital capabilities such as digital literacy and ease of use were not directly measured, limiting the ability to clearly establish causal relationships between digital competency and SMBP compliance. Third, because the study was conducted in a single rural area (Pyeongchang-gun) and included only those who owned smartphones and voluntarily agreed to participate in digital health services, the findings may have limited generalizability to a broader chronic disease population.

In addition, although the mobile application provided weekly educational content and participants who persistently exhibited abnormal readings or irregular data entry were contacted via text messages or phone calls for additional support and follow-up, detailed data on the utilization of these features were not systematically collected or incorporated into the analysis. As a result, this study was unable to evaluate potential associations between engagement with educational content or additional follow-up support and home blood pressure self-monitoring behaviors. Further analyses incorporating detailed intervention process indicators would be required to better understand the contribution of these components to sustained SMBP adherence.

Nevertheless, this study provides meaningful foundational evidence by empirically identifying factors associated with SMBP in a digital health environment and expanding the discussion on the intersection of digital and health disparities. Future research should build on these findings by quantitatively assessing digital literacy and examining the long-term clinical implications of regular SMBP adherence—such as improvements in blood pressure control and prevention of complications—across diverse regions and populations.

## Conclusion

This study examined self-monitoring behaviors and their associated factors by using a digital chronic disease management service. The findings revealed that individuals from socioeconomically vulnerable groups, such as women, those without a spouse, and those with a lower income and from health-vulnerable groups, such as current smokers and those with poor self-rated health, were more likely to exhibit irregular self-monitoring. Additionally, participants residing farther from the Smart Health Management Center showed lower monitoring adherence, highlighting that physical access to support remains a critical factor, even in digital healthcare environments.

These results underscore that individuals in greater need of health management may also face greater challenges in utilizing digital health tools and services. Therefore, the successful expansion of digital healthcare services requires tailored education and support for vulnerable populations as well as complementary systems to ensure adequate physical support infrastructure and improve digital literacy.

## Supplementary Information

Supplementary Material 1.

## Data Availability

The datasets used and/or analyzed during the current study are available from the corresponding author on reasonable request.
