# Supplementary material for: Determinants of regular self-monitoring of blood pressure in a digital health-based management program among Korean adults living in a remote community : a community-based observational study
Source: BMC Public Health. 2026 Apr 15;26:1693. doi: 10.1186/s12889-026-26974-5 (PMC13200493; doi:10.1186/s12889-026-26974-5)
Supplement: Supplementary file 1 — Supplementary Material 1. [file 12889_2026_26974_MOESM1_ESM.docx]

- **Supplement 1**

**Table S1 Analysis of Maintenance and Changes in Self-Monitoring Patterns**

| **Analysis Category** | **Blood Pressure (n=115)** |
| --- | --- |
| **Pattern Distribution, n (%)** | |
| Continuously Regular*(≥ 1/week) | 41 (35.7) |
| Regular to Irregular* | 16 (13.9) |
| Irregular to Regular | 10 (8.7)† |
| Continuously Irregular(< 1/week) | 48 (41.7) |
| **Pattern Stability, n (%)** | |
| Pattern Maintenance (%) | 89 (77.4) |
| Pattern Change (%) | 26 (22.6) |
| Cohen's κ | 0.559 |
| McNemar p-value | 0.327 |
| * Regular self-monitoring was defined as performing self-monitoring at least once per week for 11 or more weeks during the 12-week period, with all others classified as irregular self-monitors.  **Pattern Maintenance = (Regular-Regular + Irregular-Irregular)/Total × 100 | |

- **Table S2** **Questionnaire items used in the present analysis**

| **Question** | **Variable type** | **Response options** | **Reference** |
| --- | --- | --- | --- |
| **Have any of your parents or siblings (parents, brothers, or sisters) ever been diagnosed with or died from any of the following conditions?** | Categorical | ① Hypertension ② Diabetes mellitus ③ Stroke ④ Other cerebrovascular diseases (e.g., transient ischemic attack, vascular dementia; men <55 years, women <65 years) ⑤ Myocardial infarction or angina pectoris ⑥ Other heart diseases (including heart failure; men <55 years, women <65 years) ⑦ None | Ministry of Health and Welfare (MOHW). Primary Care Chronic Disease Management Pilot Program Guidelines, 2020. Ministry of Health and Welfare; 2020. (in Korean) |
| **Have you ever smoked a total of at least 100 cigarettes (equivalent to 5 packs) in your lifetime?**  Standard drink equivalents: Soju 1 glass = 1 drink; Canned beer (355 mL) = 1 drink; Bottled beer (350 mL) = 1 drink; Draft beer (500 mL) = 1.3 drinks | Categorical | ① No ② Yes, currently smoking ③ Yes, but quit smoking |  |
| **Have you engaged in physical exercise during the past week?** | Categorical | ① No exercise ② Irregular exercise ③ Regular exercise (≥3 times per week) |  |
| **On average, how often do you measure your blood pressure or blood glucose at home using a self-monitoring device (blood pressure monitor or glucometer)?** | Categorical | ① 5 times per week to daily ② 2–4 times per week ③ ≤1 time per week ④ Do not measure | So HS: Testing construct validity of self-care agency scale. Seoul: Seoul National University; 1992. (Master’s thesis). |
| **How would you rate your overall health status?** | Categorical | ① Very poor ② Poor ③ Fair ④ Good ⑤ Very good | Choi YH: Is subjective health reliable as a proxy variable for true health? A comparison of self-rated health and self-assessed change in health among middle-aged and older South Koreans. Health and Social Welfare Review 2016, 36(4):431-459. |
| **Where is your current place of residence?** | Categorical | ① Pyeongchang-eup ② Mitan-myeon ③ Bangnim-myeon ④ Daehwa-myeon ⑤ Yongpyeong-myeon ⑥ Bongpyeong-myeon ⑦ Jinbu-myeon ⑧ Daegwallyeong-myeon ⑨ Other |  |
| **What is the highest level of education you have completed?** | Categorical | ① No formal education ② Did not complete elementary school ③ Elementary school graduate or did not complete middle school ④ Middle school graduate or did not complete high school ⑤ High school graduate ⑥ Technical/vocational school graduate ⑦ Did not complete college/university ⑧ College/university graduate ⑨ Graduate school or higher | - |
| **What is your current marital status?** | Categorical | ① Never married ② Married ③ Separated ④ Divorced ⑤ Widowed ⑥ Cohabiting ⑦ Other | - |
| **What is your household’s average annual income? (Including interest, pensions, and allowances)** | Categorical | ① < KRW 6 million ② KRW 6–<12 million ③ KRW 12–<18 million ④ KRW 18–<24 million ⑤ KRW 24–<36 million ⑥ KRW 36–<48 million ⑦ KRW 48–<60 million ⑧ KRW 60–<72 million ⑨ ≥ KRW 72 million | - |
| **Are you currently employed?** | Categorical | ① Yes ② No |  |
| **Date of birth** | Continuous (date) | YYYY-MM-DD |  |
| **Sex** | Categorical | ① Male ② Female |  |
